# Influence of antenatal care on birth weight: a cross sectional study in Baghdad City, Iraq

**DOI:** 10.1186/1471-2458-12-S2-A38

**Published:** 2012-11-27

**Authors:** Mohammed A Abdal Qader, Idayu Badilla, Rahmah Mohd Amin, Hasanain Faisal Ghazi

**Affiliations:** 1 Department of Community Health, Universiti Kebangsaan Malaysia Medical Centre, Jalan Yaacob Latiff, 56000 Kuala Lumpur, Malaysia; 2 United Nations University - International Institute for Global Health, Universiti Kebangsaan Malaysia Medical Centre, Jalan Yaacob Latiff, 56000 Kuala Lumpur, Malaysia

## Background

Antenatal care is defined as the care given to mothers and their fetus during pregnancy. There are many aspects of antenatal care that need to be emphasized to ensure good delivery outcome. One important outcome is low birth weight which has been defined by the World Health Organization (WHO) as weight at birth of less than 2.500 grams (up to and including 2,499 g) irrespective of gestational age. This paper aims to show the association between antenatal care and birth weight.

## Materials and methods

A cross-sectional study involving 225 newborns recruited randomly from four General Hospitals in different areas of Baghdad were carried out in 2009. Mothers of these infants were interviewed within 24 hours after delivery. Severely ill mothers, preterm deliveries and congenital malformation babies were excluded from the study.

## Results

The percentage of low birth weight babies was 21.3%. The minimum number of antenatal care visit was one, while the maximum number of antenatal care visits was 9; the mean was 3.95 ± 1.70. The percentage of mothers who attended and registered their first antenatal care during first trimester was 73.8%, as compared to 26% who registered their first visit after first trimester. There was no significant relationship between number of antenatal care visit and low birth weight babies (p = 0.89). However, time of booking and place of first antenatal visit were significantly associated (p < 0.001, < 0.01 respectively) with low birth weight.

## Conclusion

Antenatal care of the pregnant mothers is one of the important risk factors contributing to low birth weight babies. Even though the average number of antenatal visits was satisfactory, early booking at a health centre need to be properly advocated to mothers to avoid poor birth outcome such as low birth weight.

